# 

**Published:** 1914-08

**Authors:** 


### CONTENTS.

ORIGINAL COMMUNICATIONS—

Post-Operative Abnormal Adhesions, by J. O. Kinard, M. D. ........ 195

The Present Status of Gall-Bladder Surgery, by R.R. Kime, M. D., F. A. C. S., Atlahta, Ga. 198

Meeting of Fulton County Medical Society, by R. R. Daly, M. D. ........ 206

Examination of Candidates for Assistant Surgeon 210

EDITORIAL ........ 212

SELECTIONS AND ABSTRACTS ........ 213

BOOK REVIEWS ........

